# Application of a Human Factors Systems Approach to Healthcare Control Centres for Managing Patient Flow: A Scoping Review

**DOI:** 10.1007/s10916-024-02071-1

**Published:** 2024-06-18

**Authors:** Estrella Paterson, Satyan Chari, Linda McCormack, Penelope Sanderson

**Affiliations:** 1 School of Psychology, The University of Queensland, Brisbane, Australia; 2 School of Business, The University of Queensland, Brisbane, Australia; 3 Clinical Excellence Queensland, Queensland Health, Brisbane, Australia; 4 School of Clinical Medicine, The University of Queensland, Brisbane, Australia

**Keywords:** Patient flow coordination, Healthcare control centre, Risk management, Human factors, Systems approach, Scoping review.

## Abstract

Over the past decade, healthcare systems have started to establish control centres to manage patient flow, with a view to removing delays and increasing the quality of care. Such centres—here dubbed Healthcare Capacity Command/Coordination Centres (HCCCs)—are a challenge to design and operate. Broad-ranging surveys of HCCCs have been lacking, and design for their human users is only starting to be addressed. In this review we identified 73 papers describing different kinds of HCCCs, classifying them according to whether they describe virtual or physical control centres, the kinds of situations they handle, and the different levels of Rasmussen’s [1] risk management framework that they integrate. Most of the papers (71%) describe physical HCCCs established as control centres, whereas 29% of the papers describe virtual HCCCs staffed by stakeholders in separate locations. Principal functions of the HCCCs described are categorised as business as usual (BAU) (48%), surge management (15%), emergency response (18%), and mass casualty management (19%). The organisation layers that the HCCCs incorporate are classified according to the risk management framework; HCCCs managing BAU involve lower levels of the framework, whereas HCCCs handling the more emergent functions involve all levels. Major challenges confronting HCCCs include the dissemination of information about healthcare system status, and the management of perspectives and goals from different parts of the healthcare system. HCCCs that take the form of physical control centres are just starting to be analysed using human factors principles that will make staff more effective and productive at managing patient flow.

## Introduction

Over the past decade, healthcare organisations have begun to establish centralised control centres to manage patient flow, with a view to minimising delays, improving patient safety and reducing costs. Effective patient flow management—ensuring that the patient gets to the right place for the right treatment in a timely manner— is an important determinant of the quality of healthcare delivery [2]. Inefficient patient flow management is associated with emergency department (ED) overcrowding, prolonged patient wait times, treatment delays, and unsafe practices, which may result in medical error and adverse events, and contribute an increase in risk to patient safety [3–6]. Improving patient safety depends on avoiding or removing threats to patients caused by individual and systems failures, and to enhancing systems to support routine and non-routine operating conditons [7]. Despite the emergence of healthcare control centres to manage patient flow, information about their design and effect on system performance in the published literature is limited [8].

The purpose of this paper is to survey the literature on the design, function, and operation of centres for coordinating and/or controlling the flow of patients through healthcare systems, and to identify points of similarity and differences amongst such centres. Using a human factors approach, we identify the specific operational contexts of healthcare control centres, and we classify their focus according to the levels in Rasmussen’s risk management hierarchy [1].

For convenience we will describe centres managing patient flow as *Healthcare Capacity Command/Coordination Centres* (HCCCs), with the Command/Coordination option reflecting the fact that some centres exercise command whereas others coordinate without formal command. Some HCCCs are virtual in nature whereas others locate the stakeholders in a physical control room. HCCCs range in scope, from simple bed management systems [9] to call centres [10] to complex command centres that coordinate patient management across hospital systems, under any of a variety of operational situations [11, 12].

### Emergence of HCCCs

HCCCs have been established to help in managing the movement of patients through the healthcare system safely, effectively, and with the best use of resources. Modern healthcare is an intricate sociotechnical system that is considered more complex than any other industrial system [13, 14]. It comprises numerous interacting elements that include patients with diverse treatment requirements, multiple stakeholders, and complicated financial models [13]. Despite this, healthcare workers often operate in relative isolation, focusing on their own specific goals for capacity management, patient coordination, and treatment [15, 16]. This is reflected in emergency department (ED) crowding and boarding [17] and in bottlenecks that occur farther up the hospital chain, such as the unavailability of intensive care capacity for surgical patients [18, 19]. As demands on hospital services escalate due to increased case complexity and acuity, an ageing population, and as crises such as pandemics, disasters, or wars are encountered, healthcare resources and management systems become increasingly strained with negative implications for patient flow management [20–22].

It is in the above context that the need for HCCCs has arisen. The concept of a HCCC has evolved from virtual arrangements, where stakeholders across different locations within the system interact in formal and informal ways to facilitate patient coordination to specialised facilities where staff are physically co-located [8, 12, 23]. Whether virtual or physical, HCCCs are characterised by routines and rules of operation to support effective coordination.

### Human Factors Approach: Rasmusssen’s Risk Management Framework

Key to enhancing patient flow management is the application of system design principles from a human factors engineering perspective [24–27]. Human factors (HF) is the scientific discipline and profession concerned with human-system integration, with a view to improving system design and so enhancing system performance and human wellbeing. HF contributes to the design and evaluation of tasks, products, environments and systems, taking into account the properties of humans [28].

HF principles have been applied in healthcare systems contexts for over six decades. In the late 1950s Chapanis and Safren conducted a study of medication errors in hospitals [29] and since then examples of HF applications include patient safety [7, 30], medical devices [31], anaesthetics [32, 33], intensive care units [34], and emergency departments [35]. With the increased complexity of patient coordination and the emergence of healthcare control centres we were interested in exploring the application of human factors to their design and function. HCCCs are increasingly taking on the form and functions of more traditional control centres found in other safety critical sectors such as energy and transportation [36, 37]. While HF has made crucial contributions to the scoping, design, standardisation, implementation and refinement of control centres in such sectors, it is unclear to what degree HF guidance, frameworks, methods, and expertise have influenced the evolution of HCCCs.

**Fig. 1:**
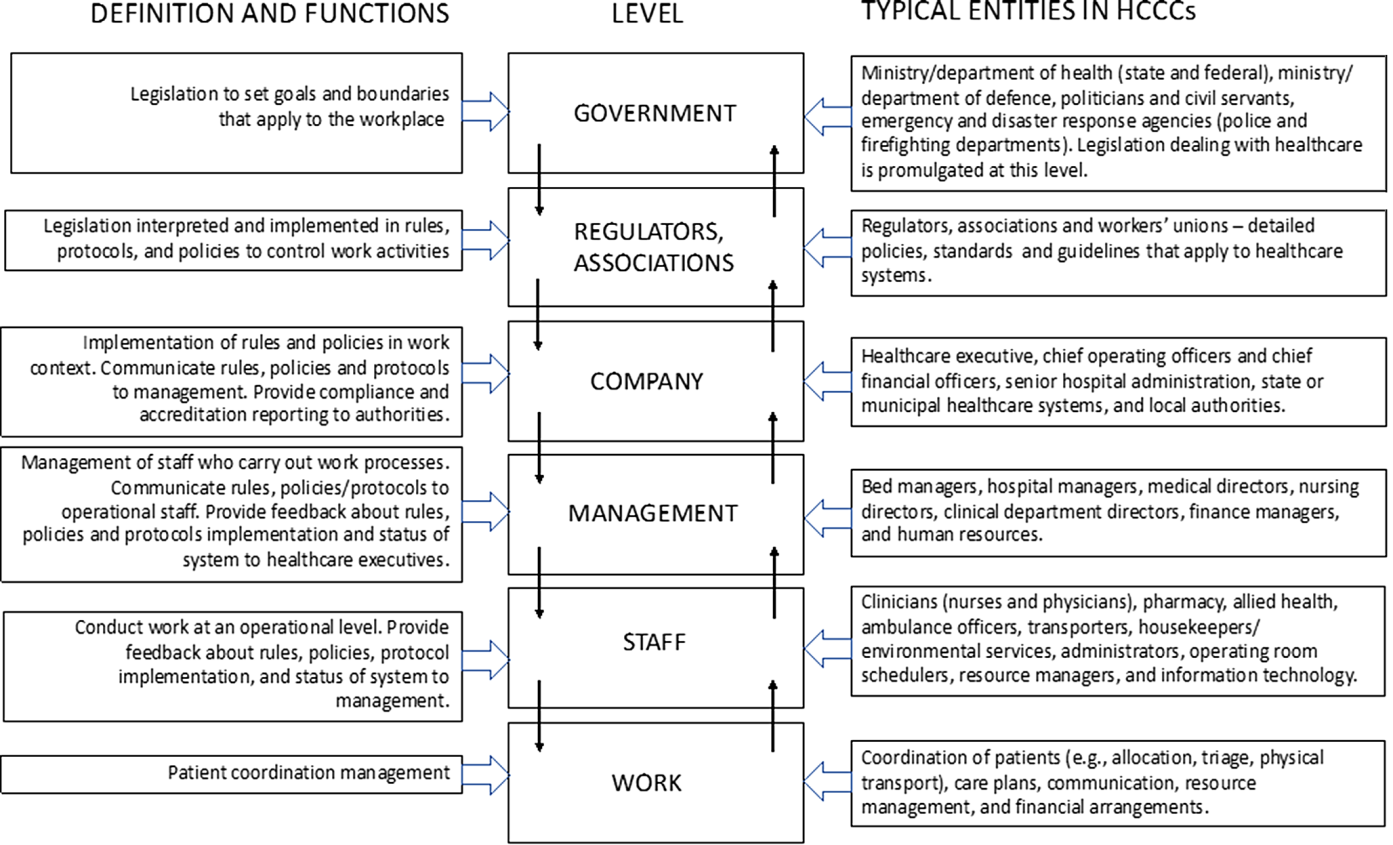
Rasmussen’s (1997) risk management framework, supplemented with definitions of each level (left) and typical entities at each level in HCCCs (right)

We adopted Rasmussen’s [1] risk management framework (RMF) to characterise the focus of papers describing HCCCs. The RMF is a widely-used framework within human factors engineering that presents a *systems perspective* on how risk is controlled in an area of activity—its levels and functions are shown in Fig. 1. Vicente adapted the RMF to the management of patient safety, using the organisational levels of patients (work), providers (staff), departments and managers (management), hospital executive and leadership (company), regulators and associations, and government [24]. Governance concerns and decisions from higher levels (control functions) about targets should be disseminated down the structure, and information about the current state of the system (feedback) should propagate up the hierarchy in an iterative manner [1, 24]. Communication is a key factor in the model and decisions made within a sociotechnical system have implications for safety. As health systems become more complex, the management of patient flow is driven by rapid development in technology, which may be faster than the pace of change in managerial structures, and legal and regulatory requirements. The fast-paced change in information and communication technologies may lead to a high degree of organisational coupling, and decisions in one element of the system may have unintended and hazardous consequences in other parts of the system [1].

As shown in the right column of Fig. 1, HCCCs operate at and across different organisational levels of complex, adaptive, sociotechnical systems. Although interaction between stakeholders within and across levels of the RMF is necessary for everyday functioning, integration across multiple levels is particularly important if a system is to address unusual challenges. Healthcare systems require effective ways to manage business as usual, but also need to handle demand surges, disasters, and extreme events of different kinds, all of which require coordination between entities and agencies at different levels of the healthcare hierarchy. Control centres have been established to facilitate such activities. As will be seen, we consider the operational focus of individual HCCCs in terms of the RMF levels they address.

### Objective of Paper

To date there has not been a comprehensive review of the broad range of HCCCs that exist. A review by Franklin and colleagues [12] has covered HCCCs with a focus on how benefits of such centres might be measured. However, they reviewed research up to 2019 across only 8 reports and since then the field has grown. A more recent narrative review surveyed 18 reports of the impact of command centres and smaller-scale centralised operating systems on healthcare capacity, from the perspective of business as usual [8]. Our review takes a broader systems perspective to investigate the following: (1) the concept of virtual and physical control HCCCs, their operational focus, and their situation in terms of the RMF, (2) existing approaches to the design and operation of HCCCs, and (3) identification of human factors issues that might be applied to HCCCs operations and that may offer opportunities for development of further guidelines for HCCC design and operation.

## Methods

We conducted a scoping review to investigate existing literature regarding patient management systems. Scoping reviews are useful for exploring fields where evidence is emerging in a disparate fashion [38, 39]. In collaboration with a librarian, we developed a systematic search strategy (see Appendix 1 for the full list of search terms). Databases included PubMed (Medline), Embase (Elsevier), Scopus (Elsevier), CINAHL (EBSCO) and Web of Science (Clarivate). To be included in this review, articles had to discuss patient management in any geographic location, be written in English, and be written from 2000 onwards. Articles could be experimental or quasi-experimental designs; descriptive observational studies; reviews, or editorial or opinion pieces. Articles were excluded if they reported control centres that specified specialised areas of healthcare (e.g., toxicology, cancer, substance control, radiology), or were conference abstracts. An initial search was carried out on 15 March 2022 followed by a second search on 12 December 2022 and subsequent reliance on Google Scholar alerts for any more recent papers.

The literature that met the inclusion criteria was imported into Endnote X9 (Clarivate Analytics, PA, USA). Endnote files were then imported into the Covidence systematic review software (Covidence, Melbourne, Australia) and duplicates were removed. One author (EP) screened titles and abstracts against the inclusion criteria. The same author then reviewed the full text of the identified literature, excluded any that did not meet the inclusion criteria, and extracted pertinent data to an MS Excel spreadsheet (Microsoft Corp., Redmond, WA, USA). The further author (PS) reviewed the full texts of the papers identified to ensure that extracted data met the inclusion criteria. Disagreements were resolved between reviewers through discussion, and if necessary, a third reviewer (SC) adjudicated.

We categorised healthcare control structures based on characteristics that included whether staff who managed patients were co-located (physical) or dispersed (virtual); and the context and type of operation carried out. To understand the function of control structures across the organisational levels of the healthcare system, we applied the RMF using six of its levels: work, staff/roles, management, company, regulators, and government. [1, 24] We also investigated whether human factors principles, industry standards, or other methods were applied to the HCCCs we identified.

## Results

**Fig. 2:**
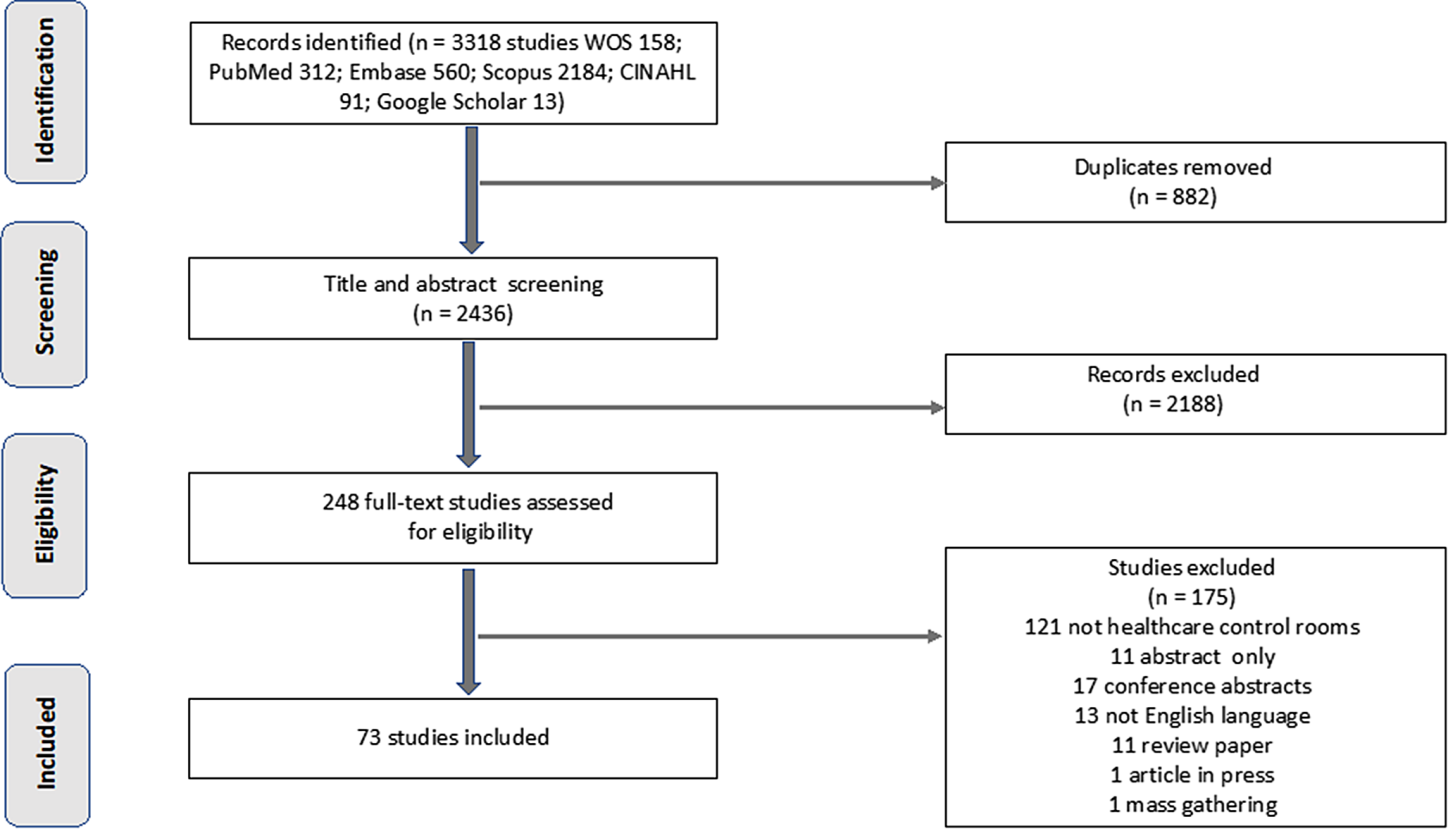
PRISMA diagram of paper selection for the review

A PRISMA diagram of the literature search and selection process is in Fig. 2. We identified 3318 articles that met the inclusion criteria. Once duplicates were removed and screening was complete, 73 articles were included in the review. Of these 73 articles, HCCCs in the USA were described in 47 (64%) of the articles, HCCCs in Canada were described in 4 articles (5%), HCCCs in Israel and Germany were described in 3 articles (4%) each, HCCCs in Korea, Taiwan, and The Netherlands were described in 2 articles (3%) each, and HCCCs Australia, Europe, India, Italy, Saudi Arabia, Sierra Leone, Sweden, Thailand, UK, and unspecified were each the subject of 1 article. It was not always possible to discern whether the HCCCs were part of public or private providers, and whether developed in house or with consultancies, so such statistics are not available.

The 73 articles selected covered two main types of patient management systems: (1) *physical control centres* where multiple staff are co-located (*n* = 52, 71%), comprising Hospital Command Centres (HoCCs) (*n* = 37, 51%) and Incident Command Centers (ICCs) (*n* = 15, 21%), and (2) *virtual control structures* where patient flow is managed by multiple staff working in separate locations (*n* = 21, 29%). We further categorised each HCCC based on its purpose (see Table 1). This resulted in four categories: business as usual (BAU) (*n* = 35), surge management (SM) (*n* = 11), mass casualty management (MCM) (*n* = 14), and emergency response (ER) (*n* = 13). Finally, we identified the organisational levels embraced by each HCCC using the properties summarized in the Typical Entities column of the RMF (see Fig. 1). The full classification of papers is in Table 2.

**Table 1:** Definitions of the categories used to discriminate the kinds of HCCCs covered in this review

Category	Description	Example
Business as Usual (BAU)	Normal, ongoing execution of standard patient flow management operations	Patient presenting to ED; patient admissions to hospitals
Surge Management (SM)	Management of increased volumes of patients due to events that exceed the normal medical infrastructure^1^ and that are ongoing.	Pandemic, infection or disease outbreak
Mass Casualty Management (MCM)	Management of increased volumes of patients due to a an overwhelming disaster event – patient needs exceed medical resources. ^2^	Natural events – earthquake, tsunami, volacanic eruption, landslide, radiation incident Manmade – terror event; bombing, explosion, mass shooting; major transport accident
Emergency Response (ER)	Management of injured or ill patient/s who require urgent medical attention.	Single vehicle traffic accident, workplace accidents; stroke; cardiac event

^1^ https://www.phe.gov/Preparedness/planning/mscc/handbook/chapter1/Pages/whatismedicalsurge.aspx

^2^ https://apps.who.int/iris/bitstream/handle/10665/43804/9789241596053_eng.pdf

**Table 2:** Coverage of risk hierarchy levels (leaving apart the lowest level, work) in the 73 papers reviewed, showing type of command center and command centre focus for patient management and patient flow. HoCC = hospital command centre, ICC = incident command center. BAU = business as usual, MCM = mass casualty management, ER = emergency response, SM = surge management. Note that within a HoCC category, a single paper many cover one or more risk management framework levels

Risk management framework level		Business As Usual	Mass Casualty Management	Emergency Response	Surge Management
PHYSICAL (52 papers)		19 (37%)	14 (27%)	9 (17%)	10 (19%)
HoCC (37 papers)		19 (51%)	4 (11%)	7 (19%)	8 (19%)
Government		3 [112-114]	4 [61, 62, 68, 72]	5 [10, 59, 97, 98, 115]	4 [49, 50, 52, 69]
Regulators/Associations		4 [9, 94, 112, 113]	4 [61, 62, 68, 72]	3 [10, 59, 115]	6 [49, 50, 52, 54, 55, 69]
Company/Hospital System		6 [40, 41, 113, 114, 116, 117]	4 [61, 62, 68, 72]	7 [10, 59, 70, 97-99, 115]	2 [49, 50]
Management		12 [9, 23, 40, 41, 43, 45, 47, 91, 112-114, 117]	4 [61, 62, 68, 72]	4 [59, 70, 99, 115]	3 [52, 54, 69]
Staff/Units		16 [9, 11, 16, 23, 40-45, 47, 91, 94, 114, 116,118]	1 [68]	5 [59, 70, 97, 98, 115]	4 [49, 55, 58, 69]
***ICC (15 papers)***		**0 (0%)**	**10 (67%)**	**2 (13%)**	**3 (20%)-**
Government		-	5 [63, 66, 67, 95, 119]	1 [74]	1 [75]
Regulators/Associations		-	7 [63, 64, 66, 67, 95, 96, 119]	2 [73, 74]	2 [56, 75]
Company/Hospital System		-	9 [60, 63-67, 71, 95, 96]	2 [73, 74]	2 [56, 75]
Management		-	9 [63-67, 71, 95, 96, 119]	2 [73, 74]	2 [56, 75]
Staff/Units		-	7 [63, 64, 66, 67, 71, 95, 119]	1 [74]	2 [56, 57]
**VIRTUAL (21 papers)**		**16 (76%)**	**0 (0%)**	**4 (19%)**	**1 (5%)**
Government		1 [48]	-	-	-
Regulators/Associations		1 [93]	-	2 [77, 80]	-
Company/Hospital System		6 [48, 76, 81, 83, 84, 89]	-	2 [77, 78]	-
Management		12 [76, 81-84, 87-90, 92, 93, 120]	-	1 [78]	1 [51]
Staff/Units		10 [48, 76, 81, 85, 86, 89, 90, 92, 93, 121]	-	4 [77-80]	1 [51]
**TOTAL PAPERS (73)**		**35 (48%)**	**14 (19%)**	**13 (18%)**	**11 (15%)**

## Discussion

In the sections that follow we discuss the properties of the physical and virtual control rooms according to the dimensions introduced above, and we consider the theoretical approaches taken to establish and operate such centres. Given that human factors issues are only just starting to be systematically addressed in the establishment and operation of HCCCs, we cover a broad range of issues relating to the human role in HCCCs that contribute to their success or otherwise.

### Physical Control Centres

The decision to co-locate patient management functions is often motivated by the perception of system-wide inefficiencies, reflected in increased times for patient admission, transfer and discharge, ED overcrowding and increased delays, increased ambulance diversions, delayed bed assignment, delays in operating room discharges, and decreased patient satisfaction [40–42].

Eight papers discuss the benefits of physically co-locating staff with respect to patient management parameters [16, 23, 41, 43–47]. For example, Kane et al. analysed the existing patient capacity in a health care system and set out to enhance patient flow by reducing patient boarding in the ED, and improving critical transfer care processes from outside facilities [41]. They created a centralised, physical command centre that helped staff streamline work, share best practices, and prioritise projects. Hospital occupancy increased from 85 to 92% and ED delays decreased from 9.7 h to 6.3 h. Further, Davenport et al. showed that the centralisation of patient transfer and flow processes across two hospitals led to a 19% increase in transfer volume, improved ICU bed availability and an annual cost saving of US$1.5M [43]. Finally, Lovett et al. showed that implementing a patient flow management centre in a multicampus academic health system improved ED metrics such as a reduced number of ambulance diversions time from ED entry to seeing a provider, environmental services turn around time (and bed assignment time) and the number of patients who departed without being seen. These improvements occurred despite increased admissions, transfers and emergency department visits, and resulted in substantial cost savings. Reduction in leaving without being seen rates was estimated to save $2.1 million per annum [40].

As Table 2 shows, the HoCCs included in the review deal principally with BAU, where the most commonly-involved organisational levels are staff/units and hospital management. Rules and routines for handling BAU naturally devolve to lower levels of the RMF. However, control rooms need to be agile to deal with a surge in patient presentation, which varies depending on circumstances such as time of day, day of the week, seasonality or surge events [48–55]. If an abnormal event leads to increased patient presentations, a HoCC can be transitioned to a surge management model. For example, one paper describes how a HoCC transitoned to handle surges in a paediatric hospital [55]. Other papers describe specific incidents, including the management of patients with infectious diseases such as COVID-19 [49, 50, 52, 54, 56–58] and Ebola [59], natural disasters such as hurricanes or tsunamis [60–67], manmade disasters such as radiation incidents [68], food borne disease outbreaks [69], mass casualty events such as terror-related events [70, 71], bomb threats and transport accidents [72]. As situations move beyond BAU to MCM, ER and SM, the involvement of higher levels of the RMF up to government itself is more firmly represented. This is to be expected when a HoCC extends its focus to more widespread or critical incidents and more agencies become involved.

At the most extreme levels, ICCs are established to deal exclusively with situations beyond BAU—mostly MCM, although we found two papers covering ER [73, 74] and three covering SM [56, 57, 75]. ICCs are usually closely coupled with outside stakeholders and agencies who manage other aspects of incident response. Key staff need to be co-located to receive timely and relevant information, communicate quickly and maintain situation awareness to enhance decision-making. Formal communication pathways and protocols across organisational levels are necessary for coordinated and efficient incident response. We found a balanced representation of organisational levels in the RMF for ICCs, as would be expected given the multi-agency coordination required for incident response.

### Virtual Control Centres

When managing patients on a BAU basis, not all hospital systems create a full-scale physical control room. This may be due to cost and resource constraints, or satisfaction with how patient flow functions are being managed virtually [23, 76]. In virtual control rooms, staff tend to be dispersed across the system and they communicate electronically, yet they have similar staff complements, goals, and tasks to physical control rooms.

As Table 2 shows, most of the virtual HCCCs included in the review deal with BAU, with a few dealing with ER [77–80] and one with SM [51]. Again, the levels of the RMF most involved in BAU are staff/units and management. Existing hospital resources can be used to improve patient flow management by improving communication about patients’ care plans and discharge needs [81]. Other initiatives include the introduction of software products such as bed management systems and electronic dashboards [81–87]; the establishment of a bed management team [86]; regular bed management/safety huddle meetings [81–85, 87, 88]; the appointment of a transfer coordinator/team [48, 82, 89]; and expedited discharge times [82]. Moreover, introducing a logistic management program with a dedicated logistics manager to coordinate patients improved patient assessment time and length of stay metrics in an ED [82].

Four papers addressed the use of a virtual control structure to manage emergency response activities relating to burn casualties [77, 80] and military patients [78, 79]. Only one paper described a virtual control structure for surge management of adult and paediatric patients with COVID-19 and the challenges of stepping up to a higher tempo of work [51]. No paper described the application of a virtual control structure for the management of mass casualty events.

### Systems-Wide and Human Factors Approaches

Several studies noted that a system-wide approach is needed to improve patient management, while taking into account competing hospital priorities [11, 41, 44, 45, 49, 55, 82, 84, 90, 91]. The articles relied on various systems-based frameworks for investigating patient management, including the concept of High Reliability Organisations (HRO) [16, 43]; lean management models [23, 83, 86, 90]; Plan-Do-Study-Act [49, 75, 85, 90]; quality improvement (QI) [83, 85, 92, 93]; process mapping/tracking [78, 81, 83, 85]; Ishikawa Diagrams [78, 83]; and Design-Measure-Analyse-Improve-Control (DMAIC) [83]. One paper described a systems engineering approach to analyse and maximise capacity in one health system for BAU, which resulted in the establishment of a centralised capacity command center [41]. Two studies detailed a systems thinking approach to optimise the response and preparedness plan for pandemics [49, 54]. In addition, we identified articles describing protocols and guidelines that relate to patient management for BAU [9, 16, 40, 41, 43, 48, 81, 84, 90, 93, 94]; mass casualty management [61, 63–66, 71, 72, 95, 96]; and emergency response [70, 73, 77, 97–99].

The HoCCs identified in this review describe some aspects that apply to the different levels of the RMF. Lovett et al.(2016) and Alhaidar et al. (2020) describe factors within the lower levels of RMF – staff and work– including staff responsible for bed management, case management, housekeeping/environmental services, patient transport and ambulance and helicopter transport [11, 40]. Kane et al. (2019) outline patient admission practices and provide information about a control centre that includes transfer operators, admission coordinators, bed managers, and internal and external transport coordinators. However, the study does not include housekeeping or inpatient transport functions [41]. Davenport et al. (2018) discuss an HRO operations centre that is more than colocation of operational staff. However, they also describe the importance of the contribution of executive management to manage patient flow effectively [43]. Lovett et al. (2016) emphasize the importance of top-down visible support from the senior executive and the need to include, educate and raise awareness of patient flow among staff across the organisation [40]. Kane et al. (2019) describe the formation of a planning workgroup that included healthcare system leadership, department directors, control centre project leaders and health technology consultants to work on the design and implementation of the new patient flow technology [41]. Collaboration of executive leadership with deparment heads that influence patient throughput across a healthcare system, including transfer centre, bed management, ambulance dispatch, staffing office, ED, operating room services and specialty care services is important for patient coordination [43, 45].

Interestingly, we found little evidence of specialised guidelines for the design, operation or evaluation of healthcare coordination centers. For example, we found no papers in this review that cited the application of industry or human factors/ergonomics standards that applied to the design, operation and evaluation of healthcare control rooms. For HCCCs established in dedicated physical facilities, the ISO11064 international standard on the ergonomic design of control centres could provide guidance on certain aspects of design [100], including a control centre’s physical layout, work stations, displays and controls; environmental and ergonomic requirements; and principles for evaluation. However, the standard emphasizes the physical aspects of the control room design rather than how demands on operators’ cognitive and social tasks are met. The standard was first published in 2000, well before the emergence of physical HCCCs, and it was intended to apply broadly across industries such as energy, resources, transportation, and aerospace [36, 100]. The complexity, variability and dynamic nature of healthcare systems produces human factors challenges that might not have been considered when the standard was written.

In general, literature describing control structures for the facilitation of patient management is diverse, and guidance on the implementation and operation of such systems is lacking [8, 11, 12, 43]. Papers such as Appelbaum et al. provide some guideance on the establishment of an incident command center to manage a healthcare crisis [75]. Moreover, market intelligence is starting to appear in the grey literature on best industry practices for the structure and functioning of HCCCs; for example, a program of investigation into the HCCC ‘ecosystem’ by KLAS Research is currently under way [101, 102].

Nonetheless, the first signs of the systemetic application of human factors principles to HCCCs have come from the work of Alhaider et al. [11] who used social network theory to represent task, knowledge, and social networks in HCCCs, and from the work of Hybinette et al. [76] applying a ‘joint cognitive systems’ approach to analyse the cognitive work of managing patient flow in a virtual HCCC context. Such examples may promote the development of further principles, guidelines, or even standards for the design and evaluation of HCCCs that take into account the cognitive and social needs of HCCC staffmembers, and that go beyond the considerations in ISO11064.

### Human Related Issues for HCCCs

#### Perspective Sharing

Within healthcare systems each microsystem has its own goals, working processes and resources, and frequently there are tensions between different departments, and between administration and frontline staff [40, 93]. A lack of appreciation of systems knowledge across all organisational levels in the RMF results in attempts to solve problems at a local level at the expense of benefits to the whole system [103]. Finding ways to enhance teamwork is critical. Co-location in a physical control room allows for opportunistic and faster communication and information sharing, and sensitivity to operations, thereby enhancing staffmembers’ overall situation awareness [11, 16, 37, 43]. It makes everyone’s work visible and can help to eliminate any “us-versus-them” mentality [23, 82]. It also shifts performance from the individual to the collective and provides a platform for the merging of collective skills, improves professional relationships, and enhances staff engagement [16, 23]. For example, promoting teamwork in intrahospital transfers showed that staff with experience or knowledge of work done in other units were able to express compassion for their colleagues [93]. Furthermore, the permanence of a physical structure to house the control room signals a concrete platform for culture change [41].

#### Information Technology – Real-Time Data and Predictive Analytics

Research indicates that information and communication technology can potentially support patient coordination by improving data dissemination processes [8, 40, 104]. A consistent theme we identified was the importance of information flow for efficient patient flow management, whether across the different elements of a healthcare system [11, 63, 66, 80, 92, 93] or between healthcare entities and outside organisations for disaster management [49, 55, 62, 69, 70, 72, 73, 95]. Collaborative communication allows staff to adapt quickly to abnormal and fast changing events such as rapidly changing patient volumes and staffing challenges [43]. Several studies describe the need to provide accurate and timely information to enhance staff members’ situation awareness, and thereby promote effective decision-making for patient management [11, 43, 64, 95]. Alhaider et al.’s [11] analysis of task, knowledge, and social networks showed the communication interdependencies between staff who managed different aspects of patient coordination, including patient transport, admission, bed assignment and housekeeping services. They suggest that the combined networks indicate situation awareness elements that should be represented in information systems to improve time to access information.

Making data available across a system enhances general awareness of patient management status and creates transparency [40]. Data need to be displayed in ways that make it easy for the user to read and extract information [40, 41, 45]. Dashboards/tiles that project integrated data feeds and provide real-time data relating to factors such as capacity management and daily workflow operations may provide information that allows for timely decisions. Also useful is information on historical trends on key metrics, which can help users and management understand operational issues and guide future strategies. Martinez et al. demonstrate the benefits of conveying carefully-selected key performance indicators to all stakeholders [105, 106]. A human factors consideration is that frontline staff should be included in the design of technology interfaces—appropriately designed information systems can reduce workload and stress [16, 43].

Several papers highlight the problem that data are fragmented across healthcare information systems and they emphasize the importance of integrating data to relieve staff of the cognitive burden of doing so, allowing more timely and effective decisions on patient management to be made [11, 23, 43]. For example, Kane et al. describe a healthcare organisation where data relating to patient flow were distributed across seven unique systems that were not fully interactive, making it difficult for staff to access information [41]. Further challenges with information dissemination include avoiding data overload, determining the currency of data, and ensuring the correct organisational processes are in place to facilitate information transfer [23, 107]. Mistrust can arise when data is entered manually and inaccuracies become evident [23].

Healthcare is a data-rich environment and articifial intelligence and machine learning algorithms can be integrated into systems to predict current and short-term future state of the system to manage patient coordination and resource allocation. Predictive analytics can be used for bed calculator applications [8, 16, 41]. Salehhnajed and Proudfoot (2023) describe more advanced predictive applications – the implementation of a healthcare command centre developed with collaboration between healthcare consultancies, clinicians, and data scientists, and which uses algorithms for complex data integration to provide real-time data, status predictions and prescriptive suggestions tailored to end users’ needs [108] They outline the use of digital twin control centres that allow for real-time data delivery, as well as predictive functions that help frontline practioners care for patients and executives manage organisational risks.

Such technologies are scalable and have the potential to have a multiplier effect. Once developed and tested they could be replicated throughout the healthcare system and by automating data tasks they may reduce human input, thus allowing more time for patient care [108]. Although automated technologies have the potential to improve system performance, concerns exist such as its effects on operator performance and change in operator roles, skill degradation and explainability/interpretability of AI [109, 110]. Other considerations include investment in technology, new training needs, patient privacy, cybersecurity and regulatory and legal issues [108].

The establishment and ongoing operational success of patient management systems is an iterative process that involves sharing of stakeholders’ perspectives and industry expertise. This requires continuous top-down (control) and bottom-up communication (feedback) across organisational boundaries, as illustrated in the RMF. Clear feedback to the upper levels of the RMF about the state of the system is important [24]. Many papers noted how important visible support from the management and organisational leadership levels in the healthcare system is for the success of an HCCC [23, 24, 40, 41, 43, 72, 81]. Leaders were encouraged to be involved in the effort to improve patient management – by visiting operation centres, attending patient flow meetings, sharing performance metrics, and responding when barriers to patient flow are identified [81]. Such involvement promotes integrated, inclusive and timely decision-making [16, 23].

### Limitations

There are several limitations to this review. First, some of the literature is of only modest quality. However, the aim of the review was to assess the literature for information about healthcare control rooms in broad terms. Second, relevant papers may have been missed because of the wide scope of the review. Third, we did not review grey literature, which may have provided further perspectives and examples. Fourth, an important emerging topic is the sophisticated technology systems that use AI and machine learning to manage the care and coordinationof patients through the healthcare system. Technology exists for remote patient monitoring, predicting patient deteriorisation, illness and outcomes, activating early warning systems, and resource allocation. A full accounting of such developments was beyond the scope of our review but limited accounts can be accessed within the literature [8, 108, 111] Despite these limitations, the review identifies factors related to the function of healthcare control rooms for managing patient flow and provides a starting point for further research.

## Conclusion

Control centers for managing patient flow are becoming more prevalent across healthcare settings. The evidence from this review provides some understanding of the operation of HCCCs and highlights the importance of a systems approach to patient flow management and the relevance of input from stakeholders and agencies across the system. The risk management framework provides a respresentation of the complex healthcare system and illustrates the gap between policy makers who propagate control functions and frontline workers who implement them. As a discpline examining the role of humans in complex sociotechnical systems, human factors could be contributing more to the design, operation, and evaluation of HCCCs. As noted above, communication and information flow across all levels of the system are essential for effective patient flow management. Human factors studies can offer analyses of the format and flow of information, and recommend effective solutions. In addition co-location of staff has the potential to break down professional silos, clarify authority structures, enhance transparency and trust, and to provide a place where staff can share information so that goals can be aligned. Human factors studies can offer analyses of team structures and communications practices to make the most of the resources offered. Substantial investment is required to put systems in place that support better patient flow management while increasing efficiency and cost effectiveness. If appropriate attention is given to supporting the human agents in HCCCs, such centers also have the potential to improve patient care and safety, and staff well-being, to reduce financial costs, and ultimately promote population health.

## Appendix 1: Literature Search Terms

PATIENT FLOW MANAGEMENT” OR “BED MANAGEMENT” OR “BED CAPACIT*” OR “PATIENT FLOW” OR “PATIENT TRANSFER*” OR “PATIENT TRANSITION*” OR “PATIENT TRANSPORT*” OR “PATIENT JOURNEY” OR “PATIENT CARE PROCESS” OR “PATIENT ACCESS” OR “PATIENT COORDINATION” OR “PATIENT CAPACITY.

AND

CONTROL CENTER” OR “CONTROL CENTRE” OR “CONTROL ROOM” OR “COMMAND CENTER” OR “COMMAND CENTRE” OR “COORDINATION CENTRE” OR “COORDINATION CENTRE” OR “MEDICAL CENTRE” OR “MEDICAL CENTER” OR “MANAGEMENT CENTRE” OR “MANAGEMENT CENTER” OR “OPERATIONS CENTRE” OR “OPERATIONS CENTER” OR “COORDINATION HUB.

AND.

HOSPITAL OR “HEALTHCARE” OR “HEALTH CARE” OR “HEALTH SERVICE” OR “HEALTH SYSTEM”.

NOT “POISON” OR “INTOXICATION” OR “DRUG” OR “CANCER”.

Inclusion criteria – HEALTHCARE CONTROL ROOM/ ANY LOCATION/ENGLISH LANGUAGE.

Exclusion criteria – TOXICOLGY/DRUG/SUBSTANCE/TOBACCO/CANCER/NOT ENGLISH LANGUAGE/NOT HEALTHCARE CONTROL ROOM/CONFERENCE ABSTRACT/ABSTRACT ONLY.

## Data Availability

Not applicable.
